# The efficacy and safety analysis of first-line immune checkpoint inhibitors in pulmonary sarcomatoid carcinoma

**DOI:** 10.3389/fimmu.2022.956982

**Published:** 2022-10-31

**Authors:** Zhimin Zeng, Xiaoying Qian, Fanrong Liu, Yong Wang, Yong Yuan, Chen Fang, Xinwei Zhang, Shangkun Yuan, Renfang Chen, Biao Yu, Tong Wang, Yan Yin, Yong Li, Anwen Liu

**Affiliations:** ^1^ Department of Oncology, The Second Affiliated Hospital of Nanchang University, Nanchang, Jiangxi, China; ^2^ Jiangxi Key Laboratory of Clinical Translational Cancer Research, Nanchang, Jiangxi, China; ^3^ Radiation Induced Heart Damage Institute of Nanchang University, Nanchang, Jiangxi, China; ^4^ Department of Medical Oncology, The First Affiliated Hospital of Nanchang University, Nanchang, China; ^5^ Medical Innovation Center, The First Affiliated Hospital of Nanchang University, Nanchang, China; ^6^ Department of Pathology, The Second Affiliated Hospital of Nanchang University, Nanchang, China; ^7^ Department of Thoracic Surgery, The Second Affiliated Hospital of Nanchang University, Nanchang, China; ^8^ Department of Pathology, The First Affiliated Hospital of Nanchang University, Nanchang, China

**Keywords:** pulmonary sarcomatoid carcinoma, immune checkpoint inhibitors, immunotherapy, anlotinib, camrelizumab, tislelizumab

## Abstract

**Background:**

Pulmonary sarcomatoid carcinoma (PSC) is a rare and aggressive disease without standardized treatment strategies. The efficacy of second-line or beyond immune checkpoint inhibitors (ICIs) has been proven in recent studies, whereas the evidence for first-line immunotherapy for PSC is still limited to case reports and remains poorly understood.

**Materials and methods:**

This was a multicenter, retrospective analysis of 21 patients with a histological diagnosis of PSC who received ICI as first-line therapy from January 2019 to March 2022. The expression of PD-L1 was evaluated by immunohistochemistry (IHC) using the monoclonal antibody 22C3. Low and high PD-L1 expressions were defined using the tumor proportion score (TPS), with cutoffs of 1 and 50%, respectively.

**Results:**

All eight patients had PD-L1 positivity who underwent PD-L1 expression assessment, and six patients (6/8, 75.0%) had high PD-L1 expression. Among the 21 PSC patients, seven received tislelizumab, six received camrelizumab, four received sintilimab, three received pembrolizumab, and one received durvalumab. Among them, 18 PSCs received combination therapy, whereas another three PSCs received immunotherapy alone. Out of the 21 PSC patients, 12 (57.1%) achieved a partial response (PR), and five patients had stable disease (SD) as the best response, whereas four PSCs experienced dramatic progressive disease (PD). The median progression-free survival (PFS) was 9.2 (95% CI [4.3, 14.1]) months, and the median OS was 22.8 (95% CI [4.0, 41.5]) months. Among the three treatment groups (immunotherapy alone, immunotherapy combined with anlotinib, and chemoimmunotherapy), the median PFS was 8.0, 9.4, and 9.6 months, and the median OS was 19.0, 22.8, and 30.6 months, respectively. There was no difference in PFS and OS between the three treatment regimen groups (*P* = 0.86 and *P* = 0.34, respectively) and different immunotherapies (*P* = 0.10 and *P* = 0.23, respectively). No serious adverse events (grade ≥ 3) were noted.

**Conclusion:**

First-line immunotherapy has promising therapeutic potential in the treatment of PSC. More studies are warranted to confirm these findings.

## Introduction

Pulmonary sarcomatoid carcinoma (PSC), a rare and aggressive disease, accounts for less than 1% of non-small cell lung cancer (NSCLC) (1). It often is at advanced stages during diagnosis and is highly heterogeneous (2). No standardized treatment strategies exist for PSC, and conventional chemotherapy also has limited efficacy (3). In addition to potential targeted molecular therapy from genetic sequencing, immune checkpoint inhibitors (ICIs) using programmed cell death 1 (PD-1)/PD-L1 antibodies are considered to be one of the most promising immunotherapy strategies (4–7).

Studies have shown higher frequencies of genetic mutations and PD-L1–positive expression in PSC than in conventional NSCLC, and PD-L1 positivity might lead to survival benefits from immunotherapy or even a favorable response in those harboring actionable mutations (4, 8, 9). Immunotherapy by immune checkpoint blockade is emerging, and the efficacy of second-line or beyond immunotherapy for PSC has been proven in recent studies (10, 11). Furthermore, several studies suggested the remarkable response of PSC to first-line immunotherapy, while the reports are limited to the case (12, 13).

The low number of patients who have undergone immunotherapy as a first-line treatment strategy makes it challenging to evaluate the specific safety and efficacy of first-line immunotherapy for PSC. The purpose of this paper is to report the largest study of first-line immunotherapy for PSC treatment to date.

## Materials and methods

### Study design and patients

A multicenter, retrospective study was conducted at two tertiary medical institutions in the Nanchang region of China, namely The First and Second Affiliated Hospital of Nanchang University in China. The patients diagnosed with advanced PSC (III/IV) who received first-line immunotherapy from January 2019 to March 2022 were enrolled. Patients with pulmonary interstitial disease, systemic immunosuppression, autoimmune disease, or second primary malignancy were excluded from this study. Written informed consent was not required, as this was a retrospective review study. The last follow-up time was 30 April 2022. Each patient’s relevant clinical data were collected from hospital electronic medical records, including sex, age, smoking status, Eastern Cooperative Oncology Group (ECOG) score, histology, clinical stage, and distant metastasis. The best tumor response was evaluated according to RECIST version 1.1. Progression-free survival (PFS) was the time from the date of immunotherapy to the date of disease progression or death. Overall survival (OS) was the time from the date of immunotherapy to death from any cause or the last follow-up.

### PD-L1 expression

The expression of PD-L1 protein was evaluated by immunohistochemistry (IHC) performed on 4-µm formalin-fixed paraffin-embedded (FFPE) tissue sections using a Dako PD-L1 22C3 pharmDx kit (Dako, Carpinteria, CA). PD-L1 protein expression was determined by using the tumor proportion score (TPS), and the cutoffs for low and high expressions were 1 and 50%, respectively (14).

### Statistical analysis

Kaplan–Meier curves were drawn to analyze the survival of PSC patients. The corresponding 95% confidence interval (95% CI) was calculated. Statistical tests were performed in IBM SPSS version 25 (IBM Corp, Armonk, NY). *p* < 0.05 was considered statistically significant.

## Results

### Clinical characteristics

In the end, 21 PSC patients who were treated with first-line immunotherapy were enrolled. The clinical characteristics are summarized in **Table 1**. The median age was 65 (range: 39–95) years, and all the men had a history of smoking (15/21, 71.4%). In five patients (23.8%), the tumors were located in the left lung, whereas most of the tumors (76.2%) were in the right lung. All patients with PSC were diagnosed at advanced stages (III/IV). The majority of these were already in the IVB stage (61.9%) and had distant metastasis when they were diagnosed. Bone was the most common site of metastases (33.3%). Eleven out of 21 patients (52.4%) with good physical status had an ECOG performance status score of 0. Not surprisingly, pleomorphic carcinoma (PLC) was the most common pathological type of PSC at 80.9%, three cases were spindle cell carcinoma (SCC), and another case was carcinosarcoma (CS).

**Table 1:** Clinical characteristics of PSC patients.

Characteristics	No. of patients (%)
Median age (range)	65 (39–95)
Gender	
Men	15 (71.4)
Women	6 (28.6)
Smoking status	
Never	6 (28.6)
Former/current	15 (71.4)
Primary location	
Left	5 (23.8)
Right	16 (76.2)
ECOG at diagnosis	
0	11 (52.4)
≥1	10 (47.6)
Histology	
PLC	17 (80.9)
SCC	3 (14.3)
CS	1 (4.8)
Clinical stage	
IIIA–IVA	8 (38.1)
IVB	13 (61.9)
Distant metastasis	
Bone	7 (33.3)
Adrenal	5 (23.8)
Liver	5 (23.8)
RLN	5 (23.8)
Other^*^	3 (14.3)

^*^Including duodenum and pancreas.

ECOG, Eastern Cooperative Oncology Group; PLC, pleomorphic carcinoma; SCC, spindle cell carcinoma; CS, carcinosarcoma; RLN, retroperitoneal lymph node.

**Figure 1:**
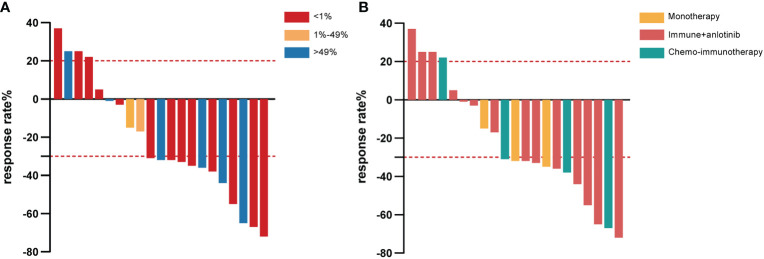
Waterfall plot of the best response rate according to PD-L1 expression in tumor cells **(A)** and different immunotherapy strategies **(B)** among PSC patients who received first-line immunotherapy treatment. PD-L1, programmed cell death ligand-1; PSC, pulmonary sarcomatoid carcinoma.

PD-L1 expression was assessed by IHC in the eight samples available. Six patients (6/8, 75.0%) had high expression (TPS > 49%) and the others had low expression (TPS 1–49%) (**Figure 1A**). Genetic testing was performed on 16 patients. Only one patient had an actionable mutation, for BRAF V600E. No EGFR, ALK, or MET mutations were found.

### Treatment and outcome of immunotherapy

Immunotherapy was given as a first-line treatment in all PSC patients, and the related data are presented in **Table 2**. The majority (18/21, 85.7%) were given combination therapies. The other three received camrelizumab (2/12, 16.7%) or sintilimab (1/12, 8.3%) monotherapy.

**Table 2:** Characteristics of first-line immunotherapy.

Characteristics	No. of patients (%)
PD-L1 expression	
<1%/undetected	13 (61.9)
1–49%	2 (9.5)
≥50%	6 (28.6)
Immunotherapy strategies	
Monotherapy	3 (14.3)
Immune+ anlotinib	14 (66.7)
Chemoimmunotherapy	4 (19.0)
Type of immunotherapy	
Tislelizumab	7 (33.3)
Camrelizumab	6 (28.6)
Sintilimab	4 (19.0)
Pembrolizumab	3 (14.3)
Durvalumab	1 (4.8)
Best response	
PR	12 (57.2)
SD	5 (23.8)
PD	4 (19.0)
Adverse events	
No	16 (76.2)
Yes	5 (23.8)

ECOG, Eastern Cooperative Oncology Group; PR, partial response; SD, stable disease; PD, progressive disease.

Among those with combination treatment, 14 were given anlotinib, a multitargeted tyrosine kinase inhibitor, combined with immunotherapy, including tislelizumab (7/21, 33.3%), camrelizumab (4/21, 19.0%), sintilimab (2/21, 9.5%), and pembrolizumab (1/21, 4.8%). Of the seven patients who received tislelizumab combined with anlotinib treatment, two achieved partial response (PR) as the best response, one of them with tumor shrinkage of approximately 72%, and four achieved stable disease (SD), whereas rapid progression occurred in another patient for PFS of 1.7 months and OS of 5.4 months (**Figure 1B**). All patients who received camrelizumab combination treatment achieved PR as their best response and a survival time of more than 14 months (**Figure 1B**). It is noteworthy that one female PSC patient harboring the BRAF V600E mutation at the IVB stage refused dabrafenib plus trametinib treatment because it was expensive, received camrelizumab combination treatment, had a PFS of 22.4 months, and is still alive at the time of this last follow-up. In another female case with TP53 mutations, the lesions suggested a PR that endured for more than 14.8 months without progression. The PFS was 8 months in the patient who accepted camrelizumab combination treatment, and the continuation of the original treatment led to sustained stability for more than 21.2 months. The last PSC patient with TP53 mutation achieved PR and had a PFS and OS of 9.4 and 22.8 months, respectively. Unfortunately, two patients with stage IVB had relatively poor physical health (ECOG ≥1), and despite treatment with sintilimab plus anlotinib, their lesion rapidly progressed and they died within 3 months (**Figure 1B**). One of them had SCC with high PD-L1 expression and KRAS mutation. A man with a smoking history at the IVB stage harboring ATM, CREBBP, KRAS, and TP53 mutation and who received pembrolizumab in combination with anlotinib achieved PR; the PFS was 10.5 months and OS was 14.2 months.

Another four patients took the combination of platinum-based chemotherapy with pembrolizumab (2/21, 9.5%), sintilimab (1/21, 4.8%), or durvalumab (1/21, 4.8%). Both patients treated with the pembrolizumab combination achieved PR (**Figure 1B**). One patient’s lesion continued remission for 11.7 months, while the PFS was not achieved. More surprisingly, the other patient had a PFS of 10 months and an OS of up to 30.6 months. A male PSC patient at the IVA stage received sintilimab combined with chemotherapy and achieved PR with tumor shrinkage of approximately 67%; the PFS was 9.6 months and the OS was not reached. On the other hand, in one patient treated with durvalumab, rapid progression occurred (only 1.8 months).

Notably, three patients were reluctant to receive chemotherapy in favor of monotherapy, two were PD-L1 positive (one with high PD-L1 expression of 95% and the other with 20%), and another did not have PD-L1 testing since there was no gene mutation. Two were treated with camrelizumab monotherapy. The OS was 12.6 months in one patient (PD-L1 95%) and more than 25.4 months in the other. Sintilimab monotherapy was adopted as a first-line treatment for one CS patient with PD-L1 expression of 20%, who had a PFS of more than 5 months and whose OS was not reached.

### Survival analysis

**Figure 2:**
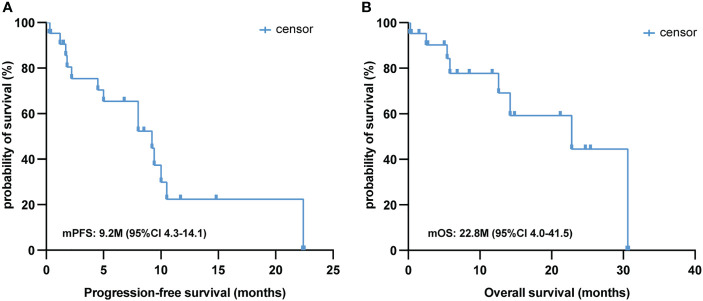
Survival analysis of first-line immunotherapy treatment in PSC. Kaplan–Meier analysis of PFS **(A)** and OS **(B)** in 21 PSC patients treated with first-line immunotherapy. PSC, pulmonary sarcomatoid carcinoma; PFS, progression-free survival; OS, overall survival.

The median follow-up time for this cohort was 8.5 (range: 0.3–30.6) months, and only seven of the 21 PSC patients (33.3%) were deceased at the last follow-up time. In the whole sample, the median PFS was 9.2 (95% CI [4.3, 14.1]) months (**Figure 2A**), and the median OS was 22.8 (95% CI [4.0, 41.5]) months (**Figure 2B**). Twelve out of 21 (57.1%) PSC patients achieved a PR and five patients had SD as the best response, whereas four PSCs experienced dramatic progress with first-line immunotherapy, although one of them was PD-L1 TPS > 50% (**Figure 1A**). Regrettably, we did not do further molecular testing or autopsies due to the family members’ refusal.

**Figure 3:**
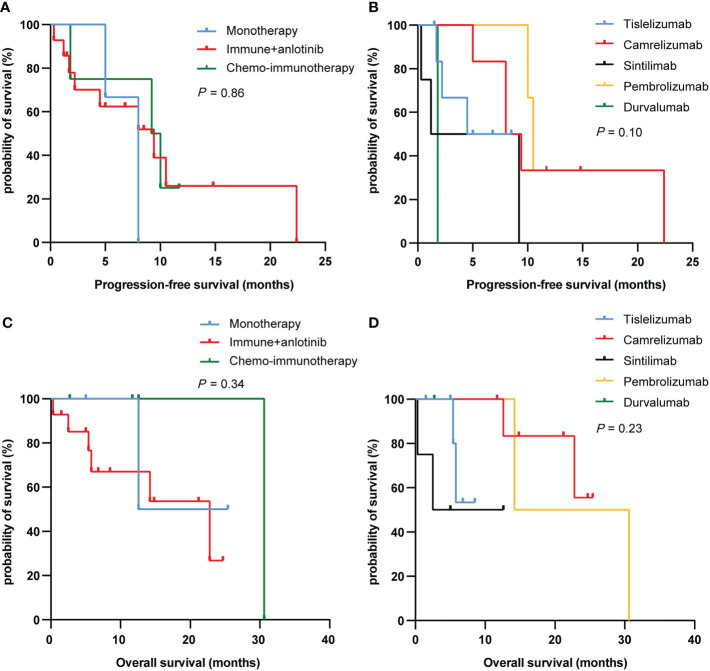
PFS **(A, C)** and OS **(B, D)** curves of PSC patients with different treatment regimens and immunotherapy. PFS, progression-free survival; OS, overall survival; PSC, pulmonary sarcomatoid carcinoma.

We further analyzed the differences between different treatment regimens and immunotherapies on PFS and OS in PSC patients. Among the three treatment groups (immunotherapy alone, immunotherapy combined with anlotinib, and chemoimmunotherapy), the median PFS was 8.0, 9.4, and 9.6 months, and the median OS was 19.0, 22.8, and 30.6 months, respectively. Nevertheless, there was no difference in PFS and OS between the three treatment regimens groups (*P* = 0.86 and 0.34, respectively; **Figure 3**). Similarly, results have shown no differences in PFS and OS between different immunotherapies (*P* = 0.10 and 0.23, respectively; **Figure 3**).

The overall objective response rate (ORR) was 57.2%, and a disease control rate (DCR) of 81.0% was achieved (**Table 2**). Remarkably, one patient who received first-line immunotherapy achieved tumor shrinkage of approximately 55%, even with the BRAF V600E mutation; the PFS was 22.4 months and the OS was not attained. We speculate that first-line immunotherapy was effective in achieving continuous remission.

### Adverse events

Five patients (23.8%) experienced adverse events (**Table 2**), including myocarditis, myelosuppression, nausea, vomiting, and diarrhea, whereas no serious adverse events (grade ≥3) were noted. None of the patients discontinued immunotherapy due to adverse effects.

## Discussion

Continuing efforts to find novel therapeutic strategies for PSC are imperative. Although the efficacy of immunotherapy after disease progression has been demonstrated (10), first-line immunotherapy in PSCs remains poorly understood. To the best of our knowledge, the present study is the largest to report the efficacy of immunotherapy for PSC as first-line immune therapy.

Immunotherapy has substantially contributed to the treatment of conventional NSCLC and has improved patient outcomes (15–19), providing promising new treatment strategies for PSC. Evidence indicates that 80.0% of PLCs with high PD-L1 expression, which is correlated with longer PFS and OS than low/negative/unknown PD-L1 expression, suggests that high PD-L1 expression may enable more benefit from immunotherapy (20). Another study showed that PD-L1 positivity in the tumor led to a better ORR of 58.8% in PSCs treated with second-line or beyond immunotherapy than PD-L1 negativity, and the median OS was 12.7 months in the whole sample. The authors thought that it was necessary to identify the efficacy of this promising immunotherapy as a first-line treatment for PSC (10).

Along with accumulating research on immune therapies in PSC, the efficacy of first-line treatment has been gradually unveiled. Pembrolizumab monotherapy treatment in three PSC patients showed a response, with a PFS of more than 11.0 months (21). Similarly, first-line camrelizumab combined with platinum-based chemotherapy, although followed by camrelizumab monotherapy for serious adverse events, achieved partial remission for more than 20.0 months (12). Interestingly, obvious tumor shrinkage was found in advanced-stage patients treated with chemoimmunotherapy, whereas PD-L1 expression was found in 1% (13). Consistent with the findings described above, our study makes significant advancements in first-line immunotherapy, including monotherapy and combination treatment.

Studies have shown that harboring the BRAF V600E mutation was associated with greater clinical benefit from ICIs and significantly prolonged PFS in NSCLC (22). Similarly, in our study, one PLC patient with the BRAF V600E mutation taking camrelizumab plus anlotinib as first-line treatment without targeted therapies had a PFS of 22.4 months. The results indicated that first-line immunotherapy is a potential choice to induce durable clinical benefits for PSC patients with the BRAF V600E mutation. Intriguingly, camrelizumab combined with anlotinib as first-line treatment demonstrated excellent effects in four PLC patients. This promising treatment strategy has been proven by a previous study showing that anti-PD-1 treatment, including camrelizumab with anlotinib, has favorable antitumor activity even in previously treated advanced NSCLC (16, 23).

Disease progression is inevitable, and little is known about what contributes to dramatic progress without any response to immunotherapy; however, we cannot ignore the four patients who showed progress in our study with advanced IVB stages at immunotherapy initiation.

KRAS and TP53 mutations have been demonstrated to be associated with high PD-L1 expression in NSCLC (24–26). Furthermore, Lococo et al. indicated that PSC harboring KRAS mutations portended a dismal prognosis (27). The results of our study are consistent with those; patients harboring KRAS or TP53 mutations indeed had high PD-L1 expression, whereas one SCC patient with a single KRAS mutation experienced limited treatment efficacy, with rapid progression, in contrast with that in one PLC patient with multigene mutations, including KRAS and TP53 mutations, who obtained a PR of more than 7.2 months. Two more PLC patients with TP53 single mutations also achieved PR for more than 8.0 months. The reasons for this are not well known; limited by a small sample size, KRAS mutation was associated with poor efficacy from immunotherapy for PSC, which is unlikely to hold true. However, this result is controversial, as several studies have suggested that those harboring KRAS mutations had a better response to immunotherapy (5, 28).

A phase Ib trial (NCT03628521) regarding combined sintilimab and anlotinib as first-line therapy in patients with advanced NSCLC saw decent success, with a median PFS of 15.0 months (29). Another retrospective study revealed that sintilimab plus anlotinib in NSCLC patients with previous systemic treatment failure yielded a favorable response (23). However, this situation did not occur in our study: Two PSC patients who took sintilimab plus anlotinib achieved rapid progression as their best response and died within 3.0 months. Similarly, the combined use of durvalumab and chemotherapy in a male PLC patient at stage IVB exhibited unsatisfactory results, although this combination of treatment strategies showed a promising effect in a previous investigation (30).

The limitations of this study are its small sample size, and we did not find an exact predictive factor or mechanism of dramatic progression. Additionally, this study had different treatment regimens and several immunotherapies with limited numbers of people on each treatment, and although the results showed no differences between groups, a larger sample of studies is needed to support this. Although remarkable outcomes have been obtained, they may suffer from selection bias (a low rate of incidence). Several clinical trials including first-line toripalimab combined therapy (NCT04725448) in the treatment of patients with advanced PSC are in progress. We hope that those studies can reveal more detail about the value of immunotherapy and help guide treatment decisions. One challenge is to be able to predict which patients are most likely to derive benefit from immune therapies and the mechanism of progression at initial treatment. Considering the rarity and complexity of PSC, clinical studies involving multiple centers, even globally, are needed to improve prognosis.

## Conclusions

Our study sheds light on the promising therapeutic potential of first-line immunotherapy in the treatment of PSC. Given these findings, a prospective study is warranted to explore the efficacy of immunotherapy with or without chemotherapy, and it is necessary to determine the reason for the dramatic progression at initial immunotherapy treatment.

## Data availability statement

The original contributions presented in the study are included in the article/supplementary material. Further inquiries can be directed to the corresponding author.

## Ethics statement

This study was reviewed and approved by the institutional ethics committee of the Second Affiliated Hospital of Nanchang University and the Medical Research Ethics Committee of the First Affiliated Hospital of Nanchang University.

## Author contributions

AL and YL co-designed the research. ZZ, XQ, and FL made a significant contribution to the data integration and analysis. ZZ provided patients’ information and data analysis. XQ drew up the manuscript. FL and YW processed the figures and tables. YYu, CF, XZ, and SY collected the outcome. RC, BY, TWand YYi followed up on the case. All authors contributed to the article and approved the submitted version.

## Funding

This study was supported by grants from the National Natural Science Foundation of China (No.81560379, 81460292, 81660315), the Surface project of the Natural Science Foundation of Jiangxi Province (No.20181BAB205046, No.20202BAB216031), Technology Supporting Program of Jiangxi Province (No.2015BBG70236), The Key Project of Education Department of Jiangxi Province (No.GJJ170012), and The Graduate Student Innovation Special Fund Project of Jiangxi Province (No. YC2022-s207).

## Conflict of interest

The authors declare that the research was conducted in the absence of any commercial or financial relationships that could be construed as a potential conflict of interest.

## Publisher’s note

All claims expressed in this article are solely those of the authors and do not necessarily represent those of their affiliated organizations, or those of the publisher, the editors and the reviewers. Any product that may be evaluated in this article, or claim that may be made by its manufacturer, is not guaranteed or endorsed by the publisher.
