# Corrigendum: The efficacy and safety analysis of first-line immune checkpoint inhibitors in pulmonary sarcomatoid carcinoma

**DOI:** 10.3389/fimmu.2023.1269596

**Published:** 2023-09-06

**Authors:** Zhimin Zeng, Xiaoying Qian, Fanrong Liu, Yong Wang, Yong Yuan, Chen Fang, Xinwei Zhang, Shangkun Yuan, Renfang Chen, Biao Yu, Tong Wang, Yan Yin, Yong Li, Anwen Liu

**Affiliations:** ^1^ Department of Oncology, The Second Affiliated Hospital of Nanchang University, Nanchang, Jiangxi, China; ^2^ Jiangxi Key Laboratory of Clinical Translational Cancer Research, Nanchang, Jiangxi, China; ^3^ Radiation Induced Heart Damage Institute of Nanchang University, Nanchang, Jiangxi, China; ^4^ Department of Medical Oncology, The First Affiliated Hospital of Nanchang University, Nanchang, China; ^5^ Medical Innovation Center, The First Affiliated Hospital of Nanchang University, Nanchang, China; ^6^ Department of Pathology, The Second Affiliated Hospital of Nanchang University, Nanchang, China; ^7^ Department of Thoracic Surgery, The Second Affiliated Hospital of Nanchang University, Nanchang, China; ^8^ Department of Pathology, The First Affiliated Hospital of Nanchang University, Nanchang, China

**Keywords:** pulmonary sarcomatoid carcinoma, immune checkpoint inhibitors, immunotherapy, anlotinib, camrelizumab, tislelizumab

In the published article, there was an error in the author list, and author Anwen Liu and Zhimin Zeng was erroneously excluded. The corrected author list appears below.

Zhimin Zeng^1,2,3†^, Xiaoying Qian^4,5†^, Fanrong Liu^6†^, Yong Wang^4,5^, Yong Yuan^7^, Chen Fang^4,5^, Xinwei Zhang^4,5^, Shangkun Yuan^4,5^, Renfang Chen^4,5^, Biao Yu^4,5^, Tong Wang^4,5^, Yan Yin^8^, Yong Li^4,5*^ and Anwen Liu^1,2,3*^

The Author contributions have also been amended to: AL and YL co-designed the research. ZZ, XQ, and FL made a significant contribution to the data integration and analysis. ZZ provided patients’ information and data analysis. XQ drew up the manuscript. FL and YW processed the figures and tables. YYu, CF, XZ, and SY collected the outcome. RC, BY, TW and YYi followed up on the case. All authors contributed to the article and approved the submitted version.

In the published article, there was an error. The approval of the institutional ethics committee of Second Affiliated Hospital of Nanchang University was not added to the ethics statement.

A correction has been made to Ethics statement. This sentence previously stated:

“This study was reviewed and approved by the Medical Research Ethics Committee of the First Affiliated Hospital of Nanchang University”.

The corrected sentence appears below:

“This study was reviewed and approved by the institutional ethics committee of the Second Affiliated Hospital of Nanchang University and the Medical Research Ethics Committee of the First Affiliated Hospital of Nanchang University”.

The authors apologize for this error and state that this does not change the scientific conclusions of the article in any way. The original article has been updated.

